# *Achyrocline satureioides* (Lam.) D.C. Hydroalcoholic Extract Inhibits Neutrophil Functions Related to Innate Host Defense

**DOI:** 10.1155/2013/787916

**Published:** 2013-02-11

**Authors:** Eric Diego Barioni, José Roberto Santin, Isabel Daufenback Machado, Stephen Fernandes de Paula Rodrigues, Viviane Ferraz-de-Paula, Theodoro Marcel Wagner, Bruno Cogliati, Matheus Corrêa dos Santos, Marina da Silva Machado, Sérgio Faloni de Andrade, Rivaldo Niero, Sandra Helena Poliselli Farsky

**Affiliations:** ^1^Department of Clinical and Toxicological Analysis, School of Pharmaceutical Sciences, University of São Paulo, Av. Prof. Lineu Prestes, 580 Bl 13B, 05508-900 São Paulo, SP, Brazil; ^2^Núcleo de Investigações Químico-Farmacêuticas (NIQFAR), University of Vale do Itajaí (UNIVALI), Rua Uruguai, 458, 88302-202 Itajaí, SC, Brazil; ^3^Department of Pathology, School of Veterinary Medicine and Animal Science, University of São Paulo, Av. Prof. Dr. Orlando Marques de Paiva 87, Cidade Universitária, 05508-270 São Paulo, SP, Brazil

## Abstract

*Achyrocline satureioides* (Lam.) D.C. is a herb native to South America, and its inflorescences are popularly employed to treat inflammatory diseases. Here, the effects of the *in vivo* actions of the hydroalcoholic extract obtained from inflorescences of *A. satureioides* on neutrophil trafficking into inflamed tissue were investigated. Male Wistar rats were orally treated with *A. satureioides* extract, and inflammation was induced one hour later by lipopolysaccharide injection into the subcutaneous tissue. The number of leukocytes and the amount of chemotactic mediators were quantified in the inflammatory exudate, and adhesion molecule and toll-like receptor 4 (TLR-4) expressions and phorbol-myristate-acetate- (PMA-) stimulated oxidative burst were quantified in circulating neutrophils. Leukocyte-endothelial interactions were quantified in the mesentery tissue. Enzymes and tissue morphology of the liver and kidney were evaluated. Treatment with *A. satureioides* extract reduced neutrophil influx and secretion of leukotriene B4 and CINC-1 in the exudates, the number of rolling and adhered leukocytes in the mesentery postcapillary venules, neutrophil L-selectin, **β**2-integrin and TLR-4 expression, and oxidative burst, but did not cause an alteration in the morphology and activities of liver and kidney. Together, the data show that *A. satureioides* extract inhibits neutrophil functions related to the innate response and does not cause systemic toxicity.

## 1. Introduction 

*Achyrocline satureioides *(Lam.) D.C. is a herb native to South America. In Brazil, it predominantly occurs in southern regions, where it is popularly known as Marcela or Macela and is largely employed in folk medicine [1]. The ethnopharmacological use of infusions prepared from inflorescences of *A. satureoides *leads to the relief of symptoms of inflammatory disorders, asthma, anxiety, gastric ulcers, and other digestive diseases [2–7]. Investigations on its chemical composition showed that the extract obtained from inflorescences is rich in flavonoids, mainly quercetin and luteolin [1, 8, 9]. 

Experimental assays* in vivo* and *in vitro* have confirmed the ethnopharmacological employment of the extracts obtained from inflorescences of *A. satureoides. *In this context, the anti-inflammatory activity of aqueous and ethanolic extracts from *A. satureoides *in carrageenan-induced rat paw edema was shown [10], which may be correlated with the modulation of the innate immune response. Puhlmann and coauthors [11] showed enhanced *in vivo* phagocytic activity of carbon particles by macrophages obtained from rats treated with *A. satureoides *extract. In addition, *A. satureoides *infusion increased peripheral blood human mononuclear phytohemagglutinin- (PHA-) induced proliferation, interferon gamma (IFN*γ*), and interleukin-4 (IL-4) secretion [6, 11]. Although the anti-inflammatory effects of *A. satureoides *have been demonstrated, its direct actions on neutrophil functions have not been shown.

Neutrophils express a wide range of membrane receptors, such as adhesion molecules and chemoattractant receptors, prompting them to react to exogenous stimuli and endogenous chemical mediators. In this context, neutrophils express the toll-like receptor 4 (TLR-4), which is responsible for the recognition of lipopolysaccharides (LPSs) from Gram-negative bacteria. LPS binds to TLR-4 on the cell membrane and activates signal-transduction pathways, mainly via MyD88, which is a central adapter protein that leads to activation of the nuclear transcription factor factor-*κ*B (NF-*κ*B). NF-*κ*B is the most important regulator of proinflammatory gene expression and induces a release of critical inflammatory molecules that are necessary to induce leukocyte migration into injured tissue and suitable immune responses [12, 13].

 Leukocyte influx to the site of the inflammatory lesion is initially dependent on interactions between circulating cells and the endothelial cells of postcapillary venules, mediated by expression/activity of adhesion molecules on the surface of both cell types. L-selectin mediates the rolling of the neutrophils along endothelial cells, as it is rapidly expressed by activated neutrophils and interacts with constitutive carbohydrates or even with P- or E-selectin expressed on endothelial cells [14]. L-selectin is cleaved, by action of metalloproteases, pointing to the leukocytes become arrested to the endothelium [15]. Therefore, integrin family molecules, especially *β*2-integrin subfamily molecules, which are mostly expressed by leukocytes, mediate firm adhesion by interacting with a diversity of endothelial membrane components and immunoglobulin superfamily molecules. Ultimately, neutrophils transmigrate between interendothelial junctions, via heterophilic and homophilic interactions of immunoglobulin superfamily molecules, such as platelet endothelial cell adhesion molecule-1 (PECAM-1), to subsequently migrate to the inflammatory focus [16]. In the extravascular matrix, neutrophils directly move to the site of the lesion in response to chemotactic chemical mediators [17]. At the lesion site, they phagocyte the lesion-causing agent and release preformed chemical substances that contribute to the destruction of the damaging agent. However, in the case of noncontrolled inflammation, blockage of phases of neutrophil mobilization into focus of the inflammatory reaction is an important therapeutic strategy. 

This study investigated the *in vivo* actions of hydroalcoholic extract obtained from inflorescences of *A. satureoides *on neutrophil trafficking from the blood into inflamed tissue. Data confirmed quercetin and luteolin to be the main constituents in the hydroalcoholic extract and showed the absence of systemic toxicity. However, results clearly showed that *A. satureoides *hydroalcoholic extract inhibits LPS-induced pathways of neutrophil migration via a mechanism that might be partially dependent on TLR-4 expression. Additional anti-inflammatory mechanisms might exist, as the extract also inhibited neutrophil activation caused by a direct intracellular stimulation of protein kinases. 

## 2. Materials and Methods

### 2.1. Chemicals

Lipopolysaccharide from *Escherichia coli *(LPS, serotype 026:B6), indomethacin, EDTA, and propidium iodide (PI) were purchased from Sigma-Aldrich (St. Louis, MO, USA). Ketamine and xylazine were purchased from Vetbrands (Paulinia, SP, Brazil), and heparin (Liquemine) was purchased from Roche Pharmaceuticals (Brazil). Anti-L-selectin-phycoerythrin (PE), anti-*β*2-integrin-fluorescein-isothiocyanate (FITC), Annexin-V-FITC, and anti-CD284/MD-2-PE (toll-like receptor 4) antibodies were obtained from BD Pharmingen (San Diego, CA, USA). Leukotriene B4 (LTB4) enzyme-linked immunosorbent assay (EIA) was obtained from GE Healthcare (Salt Lake City, UT, USA). The biochemical assays aspartate aminotransferase (AST), alanine aminotransferase (ALT), gamma-glutamyl transferase (gamma-GT), urea, and creatinine were purchased from Biotécnica (Varginha, MG, Brazil). Phorbol myristate acetate (PMA) was obtained from Calbiochem (San Diego, CA, USA), and 2′7′ dichlorodihydrofluorescein-diacetate (DCFH-DA) was obtained from Molecular Probes (Eugene, OR, USA). CINC-1 was also determined using EIA kits obtained from R&D Systems (Minneapolis, MN, USA). Air filters were purchased from TPP, Switzerland, and all reagents used in preparing the phosphate buffered solution (PBS) and ringer solution were purchased from Merck, USA.

### 2.2. Plant Material

Inflorescences of* A. satureoides *were collected in Fraiburgo, in the State of Santa Catarina, Brazil. The material was identified, and a voucher specimen was deposited at the herbarium of the State University of Maringá (UEM) with the code HUEM-23568. The material collection and all experiments were authorized by the Council of Management of Genetic Patrimony, Brazil (CGEN, process number 010062-2012-2). Air-dried plant material was cut into small pieces and macerated with 70% (v/v) aqueous ethanol at room temperature for 7 days. The macerate was filtered and the solvent removed by rotary evaporation under reduced pressure.

### 2.3. Apparatus and Chromatographic Conditions

Analysis was conducted using a high performance liquid chromatography (HPLC) system (Waters) equipped with a 600-F pump, 717 plus autosampler, followed by a line degasser (AF) and equipped with a UV-Vis detector (PDA 2996). A reverse-phase C18 column (25 cm, 4.6 mm i.d.; 0.5 *μ*m film thickness and 100A) was employed (Luna, Phenomenex) at 25°C. Chromatographic separation was performed at room temperature with a flow rate 0.8 mL/min of gradient elution using two solvents: A (aqueous methanol, 50% (v/v)) and B (water acidified with acetic acid at pH 2.3). The gradient system used was 50% A (15 min), 50%–60% A (15 min), 60%–70% A (10 min), 70%–80% A (10 min), 80%–90% A (10 min), and 95% A (5 min). UV-Vis spectra were recorded at wavelengths 200–400 nm (detection at 350 nm). Solvents used were of HPLC grade, filtered (0.2 *μ*m, Schleicher & Schuell, Maidstone, Kent, UK), and degassed by sonication before use. The samples of hydroalcoholic extract (0.54 mg/mL) and standard quercetin and luteolin (0.5 mg/mL) were dissolved in methanol and filtered through a 0.45 *μ*m membrane filter, and 20 *μ*L was analyzed in triplicate.

### 2.4. Gas Chromatography-Mass Spectrometry Analysis (GC-MS)

The GC-MS analysis was carried out using a Shimadzu Gas Chromatograph (Model QP-2010S series) equipped with an AOC-20i injector. The GC was equipped with a fused silica capillary column-TRX-1 (30 m × 0.25 mm), film thickness 0.1 *μ*m. The oven temperature was maintained at 220°C for 5 min holding time and was then raised from 220 to 300°C at a rate of 20°C/min, then held for 2 min and raised further to 310°C at a rate of 10°C/min, and once more held at this temperature for 15 min, employing helium gas (99.999%) as a carrier gas at a constant flow rate of 0.80 mL/min. Hydroalcoholic extract of *A. satureoides *(1 *μ*L) at a split ratio of 1 : 20 was injected. An MS transfer line temperature of 250°C was performed on a Shimadzu (Model QP-2010S series) coupled Gas Chromatograph equipped with an NIST08 Library software database. Mass spectra were taken at a 70 eV scanning rate of 1 scan/s. Identification of compounds was conducted using the database of the NIST08 Library. The mass spectrum of the individual unknown compounds was compared with that of known compounds stored in the software database Library.

### 2.5. Animals

Male Wistar rats (180–220 g) were obtained from the Central Animal House of the School of Pharmaceutical Sciences and Chemistry Institute of the University of São Paulo. The animals were housed in standard cages, at room temperature (25 ± 3°C), with 12 h dark/light cycles, and supplemented with food and water *ad libitum*. All procedures were performed according to the Brazilian Society of Science of Laboratory Animal guidelines for the proper care and use of experimental animals, and the experiments were approved by the local ethics committee (protocol number CEUA/FCF/334). The animals were anesthetized before each experimental procedure with ketamine/xylazine (80 : 8 mg/kg i.p.), thus preventing stress. 5-6 animals were used in each assay. 

### 2.6. Treatments

The doses used in this study were based on data previously published by Santin et al. [2], which, in turn, applied doses based on the traditional folk use of this plant. In this study, the extract was administered orally at 50, 100, and 250 mg/kg. Assays were carried out 1 or 2 hours after treatments.

### 2.7. *In Vivo* Leukocyte Migration: Air Pouch Model

Animals were anesthetized, and 10 mL of sterile air was injected subcutaneously into the dorsal region. After six days, the pouch was refilled with 10 mL air. On the tenth day following the first air injection, the animals were divided into six groups and received one of the following treatments by oral gavage: (1) sham; (2) vehicle (PBS/ethanol 10%); (3) indomethacin (30 mg/kg; positive control); or (4)–(6) *A. satureoides *(50, 100, or 250 mg/kg). After 1 h, LPS from *E. coli* (serotype 026:B6; 1 mg/2 mL PBS) was injected directly into the pouches. At 1 h or 4 h after the LPS injection, the animals were reanesthetized and sacrificed. The pouches were washed with 2 mL ice-cold PBS, and the total leukocyte number was determined using a Neubauer chamber. Differential cell counts were performed on smears stained with May-Grünwald-Giemsa.

### 2.8. Cell Viability and TLR-4 Expression by Flow Cytometry

Peripheral blood samples from male Wistar rats submitted to the air pouch model (after 1 h LPS injection) were obtained via abdominal aorta punctures. Heparin (5000 UI/mL) was used as an anticoagulant. Subsequently, the whole blood was hemolyzed (hypotonic lysis with NaCl solution at 0.2% and 1.6%) and centrifuged (5 min at 600 g) to obtain the leukocytes. The total number of leukocytes was quantified using a Neubauer chamber. Peripheral leukocytes (1 × 10^6^ cells) were incubated with Annexin V conjugated to FITC (1 : 100) for 20 min, and PI (100 *μ*g/mL) was added immediately to evaluate cell viability. To quantify TLR-4 expression, leukocytes (1 × 10^6^ cells) were incubated with anti-CD284/MD-2 (TLR-4) monoclonal antibodies conjugated to PE (1 : 100) for 60 min at room temperature. Immediately after incubations, all the samples, except for Annexin V, were centrifuged (5 min, 600 g) and resuspended in PBS for analysis in the flow cytometer FACSCalibur (Immunocytometry System, San Jose, CA, USA). Data were obtained from 10,000 cells, and only the morphologically viable leukocytes were considered for analysis. The neutrophil population was characterized by different size and complexity parameters of different cell types detected by flow cytometry. The optical signals emitted were converted into electronic signals and were analyzed by FlowJo software (Tree Star, Inc., Ashland, TN, USA). Results of TLR-4 expression are presented as fluorescence units, and apoptosis and necrosis are shown as the percentage of Annexin V- or PI-positive cells.

### 2.9. Adhesion Molecule Expression and Oxidative Burst by Flow Cytometry

Male Wistar rats (not submitted to the air pouch model) were divided into three groups and received one of the following treatments by oral gavage: (1) sham (2) vehicle (PBS/ethanol 10%), or (3) *A. satureoides *(100 mg/kg). Circulating leukocytes (1 × 10^6^) from the whole blood were collected as described in Section 2.8. To measure the adhesion molecules expression, cells were incubated with or without LPS (1 *μ*g/mL) for 60 min at 37°C. Subsequently, the leukocyte suspension was washed with 1 mL ice-cold PBS. The supernatant was discarded, and leukocytes were incubated with monoclonal anti-CD62L antibodies conjugated to PE (1 : 100) and anti-CD18 conjugated to FITC (1 : 100), for 20 minutes at room temperature. To assess the leukocyte oxidative metabolism, leukocytes (1 × 10^6^ cells) were incubated with 100 *μ*L of PMA (100 ng), 200 *μ*L of DCFH-DA (0.3 mM), and 700 *μ*L of PBS for 30 min at 37°C.

Immediately after incubations, the samples were centrifuged (5 min, 600 g) and were resuspended in PBS for quantification by flow cytometry. The neutrophil population was characterized as described in Section 2.8. Results of oxidative metabolism are expressed as units of fluorescence, and data of adhesion molecules are expressed as the percentage of positive cells.

### 2.10. Intravital Microscopy

Rats were divided into three groups and received one of the following treatments by oral gavage: (1) sham (PBS), (2) vehicle (PBS/ethanol 10%), or (3) *A. satureoides *(100 mg/kg). The rats were anesthetized 1 h after the treatment, and 30 min later, the mesentery was surgically exteriorized. Animals were maintained on a board thermostatically controlled at 37°C, which included a transparent platform on which the tissue to be transilluminated was placed. The preparation was kept moist and warm by irrigating the tissue with a warmed (37°C) Ringer-Locke solution (154 mM NaCl, 5.6 mM KCl, 2 mM CaCl_2_·2H_2_O, 6 mM NaHCO_3_, 5 mM glucose and 1% (w/v) gelatin, and pH 7.2–7.4). The rate of solution outflow onto the exposed tissue was controlled to keep the preparation in continuous contact with a film of the solution. Transilluminated images were obtained by optical microscopy (Axioplan II, Carl-Zeiss equipped with 5.0/0.30 × Plan-Neofluar or 10.0/0.25 × Achroplan longitudinal distance objectives/numeric aperture and 1.0 × 1.25 × or 1.60 × Optovar). The images were captured by a video camera (ZVS, 3C75DE, Carl-Zeiss) and were transmitted simultaneously to a TV monitor and to a computer. Images obtained on the TV monitor were recorded on software. Digitized images on the computer monitor were subsequently analyzed by image analyzing software (AxioVision). 

The interaction between leukocytes and the vessel walls was analyzed by determining the number of rolling and adherent leukocytes on the postcapillary venule wall (20–30 *μ*m diameter, 200 *μ*m length) of the mesentery. Leukocytes moving in the periphery of the axial stream, in contact with the endothelium, were considered to be rolling, and their number was determined in 10 min periods. The number of leukocytes that adhered to the endothelium (stopped at the vessel wall) was determined in the same vascular segment after 10 min. The number of rolling and adherent cells was quantified after topical application of LPS (30 *μ*g/40 *μ*L in PBS) to the venules of the mesentery microcirculation. Three fields were evaluated per animal after application. The results were then averaged for each animal.

### 2.11. Inflammatory Mediators

The air pouch lavage fluid was collected 1 h after LPS injection to evaluate the concentration of inflammatory mediators. LTB4 and CINC-1 were quantified using EIA Kits according to the manufacturer's instructions. The results were expressed as pg/mL or ng/mL, respectively.

### 2.12. Biochemical Parameters

The whole blood was collected 2 hour after treatments without anticoagulant to serum separation after centrifugation (10 min, 600 g). The concentration of kidney and liver markers was analyzed using commercial biochemical kits for urea, creatinine, aspartate aminotransferase (AST), alanine aminotransferase (ALT), and gamma-GT.

### 2.13. Histopathology

Liver and kidney samples were collected 2 hour after treatments, washed with phosphate-buffered saline (PBS), and fixed in 10% buffered formalin for 24 hours. Tissue samples were dehydrated in graded ethanol solutions, cleared in xylene, and embedded in paraffin wax. After that, serial sections (5 *μ*m) were prepared and stained with hematoxylin and eosin (H&E). Images were taken at original magnification of 100x (Eclipse E800 Microscope, Nikon, Japan).

### 2.14. Statistical Analysis

Means and the standard error of the mean (S.E.M.) for all data are presented and were compared using Student's *t*-tests or ANOVA. Tukey's Multiple Comparisons test was performed to determine the significance of the differences between experimental conditions. GraphPad Prism 4.0 software (San Diego, CA, USA) was employed. Values of *P* < 0.05 were considered significant.

## 3. Results

### 3.1. Chromatographic Analysis

**Figure 1:**
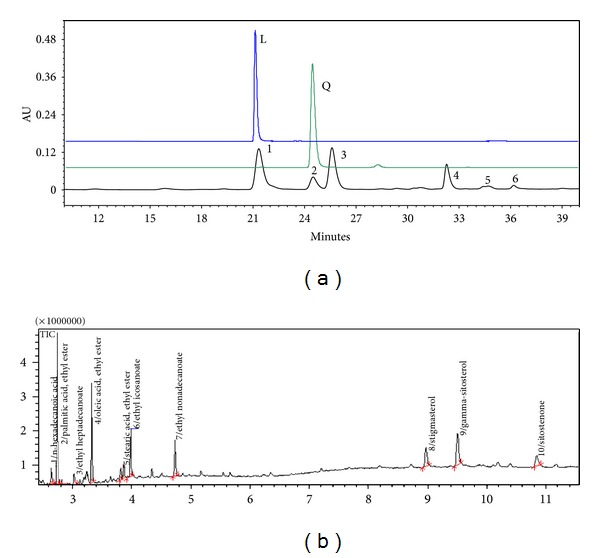
(a) HPLC overlap chromatogram profile of hydroalcoholic extract of *A. satureoides*. L: luteolin; Q: quercetin. (b) GC-MS chromatogram showing peaks of the main phytochemicals present in extract of* A. satureoides*.

The phytochemical profile of the *A. satureoides *hydroalcoholic extract showed six compounds, and two main compounds were identified (Figure 1(a)). The major components of the extract were compounds 1 and 2, which were identified by direct comparison with authentic samples and area peaks as luteolin and quercetin, respectively. Although these compounds occurred in other tissues of *A. satureoides*, the relative content of these two flavonoids from inflorescences was 12.31 and 13.65 *μ*g/mL for 1 and 2, respectively. The GC-MS analysis showed seven distinct peaks, identified via the NIST08 library software database as ethyl ester derivatives of oleic, palmitic and stearic acids. In addition, the steroids stigmasterol, gamma-sitosterol, and sitostenone were also identified in the *A. satureoides *extract (Figure 1(b)).

### 3.2. *A. satureoides* Hydroalcoholic Extract Inhibits *In Vivo* Neutrophil Migration into LPS-Inflamed Tissue

**Figure 2:**
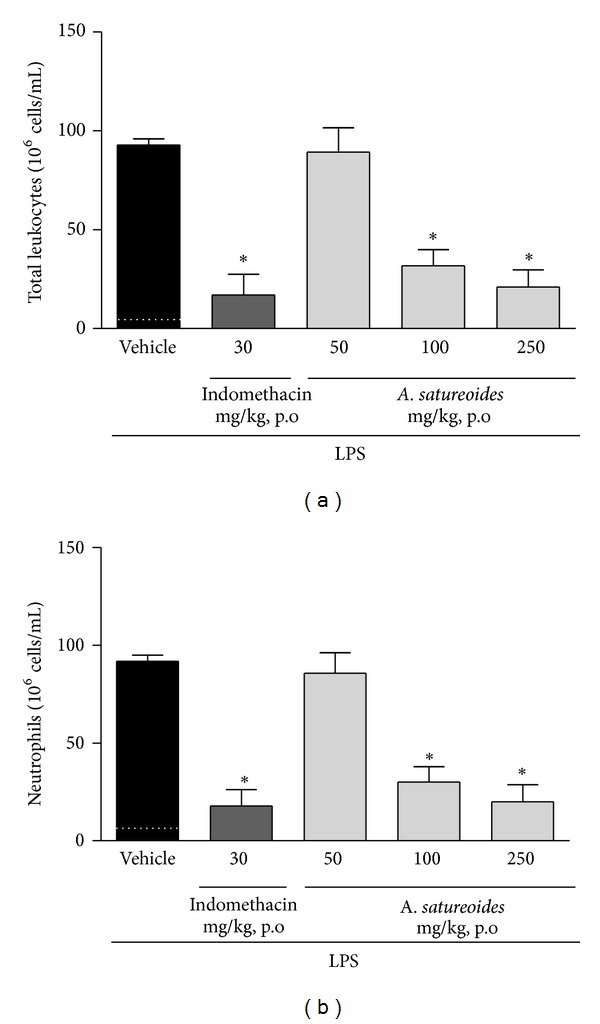
Effects of *A. satureoides* hydroalcoholic extract on *in vivo *leukocyte migration induced by LPS. Air pouch animals received orally: (1) vehicle (PBS/ethanol 10%), (2) indomethacin (30 mg/kg), or (3) *A. satureoides* (50, 100, or 250 mg/kg). LPSs from *E. coli* 026:B6 (1 mg/2 mL) or PBS were injected after 1 h directly into the air pouch, and 4 h later, the number of cells in the pouch was quantified. The dotted line indicates the number of neutrophils in the noninflamed tissue. (a) Number of total leukocytes in the air pouch. (b) Number of neutrophils in the air pouch. Data are expressed as mean ± S.E.M. of 5-6 animals in each group. Statistical analysis was performed using ANOVA followed by Tukey's test. *P < 0.05 versus vehicle.

The *in vivo *anti-inflammatory effect of the hydroalcoholic *A. satureoides *extract on LPS-induced inflammation in the subcutaneous tissue of rats (air pouch model) was investigated. Different doses were tested, and the number of cells migrating into the pouches was determined 4 h after injection of LPS or PBS. Results presented in Figure 2(a) show that* A. satureoides *treatment reduced leukocyte migration to the inflammatory site, which reflected an impaired influx of neutrophils (Figure 2(b)). The effect caused by 100 mg/Kg of *A. satureoides* extract was similar to that evoked by indomethacin treatment (Figures 2(a) and 2(b)).

Assays were conducted to evaluate the inhibitory mechanism of *A. satureoides *extract on cellular mechanisms involved in neutrophil migration to the site of the lesion. For this purpose, neutrophils were isolated from rats and incubated *in vitro* with the extract; thus, the direct action of the extract in each phase of neutrophil migration could be investigated. However, *in vitro* incubation with the extract caused cell death, especially by necrosis (data not shown). For this reason, all the following assays were conducted using blood and circulating leukocytes collected after *in vivo* vehicle or *A. satureoides *extract treatments.

### 3.3. *A. satureoides* Hydroalcoholic Extract *In Vivo* Treatment Does Not Affect Neutrophil Viability

Based on cell viability data obtained from *in vitro* studies, it was also relevant to investigate leukocyte viability from circulating blood following *in vivo *treatments with *A. satureoides *extract. Data presented in Table 1 show that extract treatment did not cause cell death, as determined by the Annexin V/PI labeled flow cytometry assay. 

### 3.4. *A. satureoides* Hydroalcoholic Extract Impairs *In Vivo* Leukocyte-Endothelial Interactions

**Figure 3:**
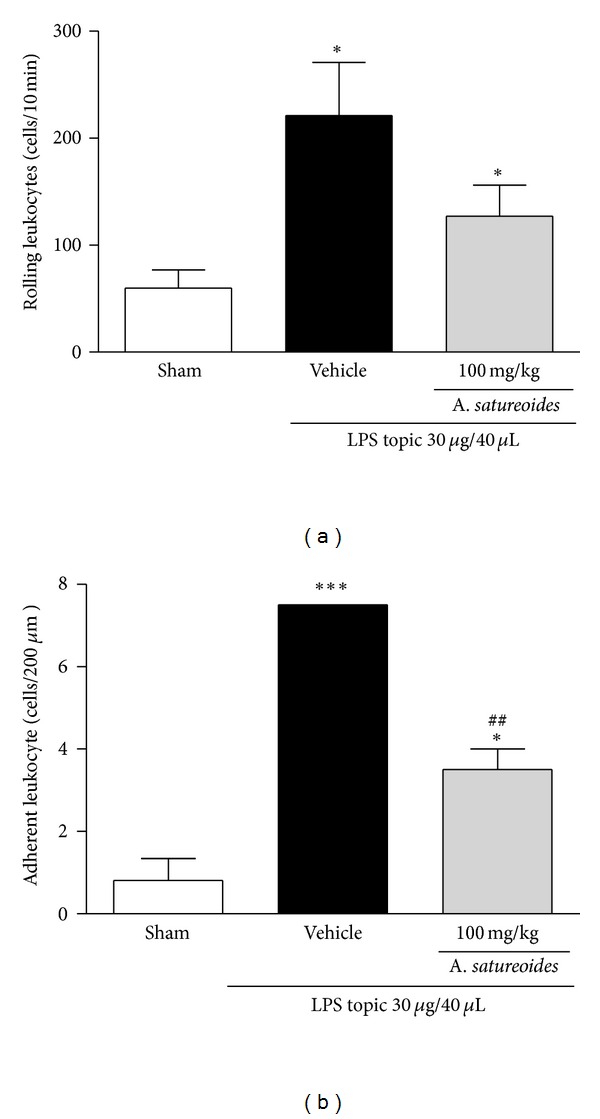
Effect of *A. satureoides* hydroalcoholic extract on leukocyte-endothelial interaction *in vivo*. Animals received orally (1) sham (surgical manipulation without treatment), (2) vehicle (PBS/ethanol 10%), or (3) *A. satureoides* (100 mg/kg). After 1 h, LPS from *E. coli* 026:B6 (30 *μ*g/40 *μ*L) was applied topically into the mesenteric network, and the number of rolling and adherent leukocytes was quantified. (a) Number of rolling leukocytes. (b) Number of adherent leukocytes. Data are expressed as mean ± S.E.M. of 5-6 animals in each group. Statistical analysis was performed using ANOVA followed by Tukey's test. *P < 0.05 and ***P < 0.001 versus sham; ^#^ *P* < 0.05 and ^##^ *P* < 0.01 versus vehicle.

The behavior of leukocytes in the peripheral blood was evaluated by direct observation of the microcirculation network. Administration of *A. satureoides *extract slightly reduced the number of LPS-induced rolling leukocytes in comparison to the number observed in vehicle-treated rats (Figure 3(a)). However, administration of the extract markedly reduced the LPS-induced adherence of leukocytes to the vessel wall of the mesentery network (Figure 3(b)). It was further shown that *A. satureoides *extract did not induce toxicological effects on the microcirculation, such as hemorrhage, thrombus formation, or vascular stasis (data not shown).

### 3.5. *A. satureoides* Hydroalcoholic Extract Alters Neutrophil Adhesion Molecule Expression

**Figure 4:**
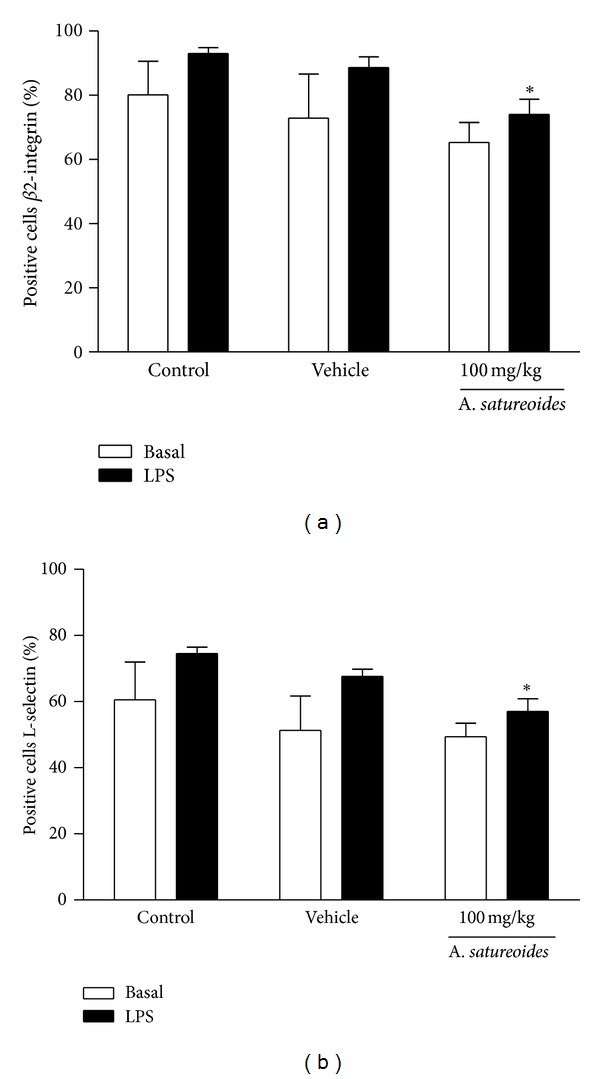
*β*2-integrin and L-selectin expression on neutrophils from animals treated with *A. satureoides* hydroalcoholic extract. Animals received orally (1) control (untreated animals), (2) vehicle (PBS/ethanol 10%), or (3) *A. satureoides* (100 mg/kg). Neutrophils were incubated with PBS or LPS from *E. coli* 026:B6 (5 *μ*g/mL) for 60 min at 37°C. (a) The percentage of *β*2-integrin-positive neutrophils and (b) the percentage of L-selectin-positive neutrophils. Data are expressed as mean ± S.E.M. of 5-6 animals in each group. Statistical analysis was performed using ANOVA followed by Tukey's test. *P < 0.05 versus respective LPS-stimulated control.

Leukocyte-endothelial interaction is mediated by adhesion molecule expression. As *A. satureoides *extract reduced the *in vivo* leukocyte-endothelial interactions, assays were performed to investigate the actions of the extract on the expression of L-selectin and *β*2-integrin on the neutrophil surface. Results show that a dose of 100 mg/kg* A. satureoides *extract reduced the number of *β*2-integrin- (Figure 4(a)) and L-selectin-positive (Figure 4(b)) neutrophils after LPS stimulation, showing that treatment with the extract affected the ability of neutrophils to express both molecules.

### 3.6. *A. satureoides* Hydroalcoholic Extract Reduces CINC-1 and LTB-4 Secretion in LPS-Induced Inflamed Exudates

**Figure 5:**
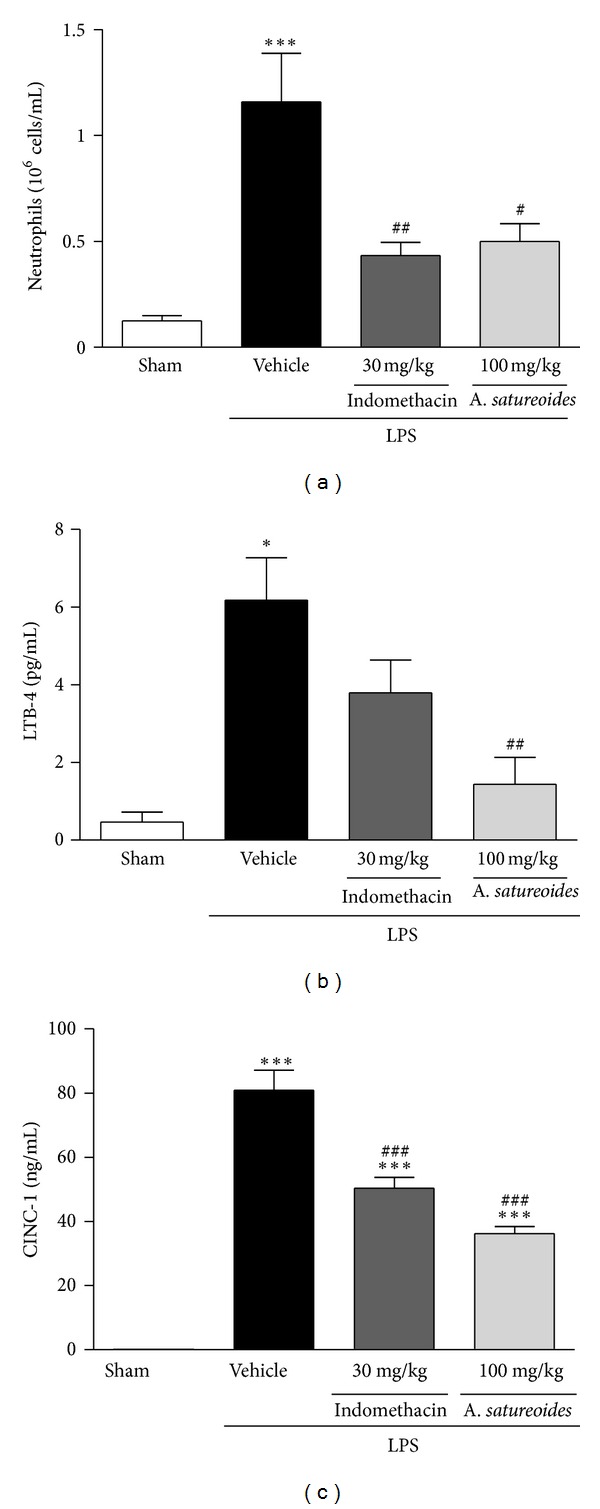
Effects of *A. satureoides* hydroalcoholic extract on *in vivo *leukocyte migration induced by LPS, LTB-4, and CINC-1 secretion. Air pouch animals received orally (1) sham (air pouch induced without treatment), (2) vehicle (PBS/ethanol 10%), (3) indomethacin (30 mg/kg), or (4) *A. satureoides* (100 mg/kg). After 1 h, LPSs from *E. coli* 026:B6 (1 mg/2 mL) or PBS were injected directly into the air pouch, and 1 h later, the number of cells in the pouch was quantified. (a) Number of neutrophils in the air pouch, (b) levels of LTB-4 on air pouch exudate, and (c) levels of CINC-1 secretion on air pouch exudate. Data are expressed as mean ± S.E.M. of 5-6 animals in each group. Statistical analysis was performed using ANOVA followed by Tukey's test. *P < 0.05 and ***P < 0.001 versus sham; ^#^ *P* < 0.05, ^##^ *P* < 0.01, and ^###^ *P* < 0.01 versus vehicle.

Chemical mediators including neutrophils are secreted in inflammatory condition by different cells. To investigate the ability of *A. satureoides *extract to inhibit the secretion of the chemotactic mediators CINC-1 and LTB-4, exudate was collected from animals treated with *A. satureoides *hydroalcoholic extract or vehicles, 1 h following LPS injection into the air pouch. This experimental strategy was employed to avoid large differences in the number of migrated leukocytes into air pouches, as observed 4 hours after LPS injection (Figure 2(b); vehicle: 92 × 10^6^; *A. satureoides*; 25 × 10^6^), which could be responsible for altered secretion of these chemokines. As shown in Figure 5(a), treatment with *A. satureoides *extract reduced neutrophil migration into the air pouch 1 hour after LPS injection (vehicle: 1.15 × 10^6^; *A. satureoides*: 0.5 × 10^6^) and decreased both LTB-4 and CINC-1 levels in the inflammatory exudate (Figures 5(b) and 5(c)). 

### 3.7. *A. satureoides* Hydroalcoholic Extract Reduces TLR-4 Expression and PMA-Induced Oxidative Burst on Neutrophils

**Figure 6:**
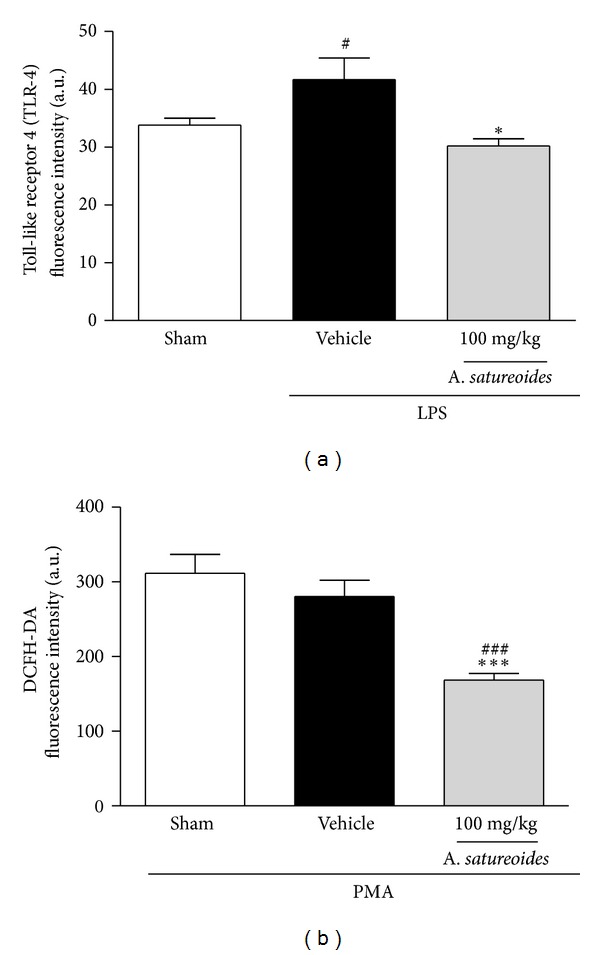
Effects of *A. satureoides* hydroalcoholic extract on TLR-4 expression and DCFH PMA-induced formation. Animals received orally (1) sham (air pouch induced without treatment), (2) vehicle (PBS/ethanol 10%), or (3) *A. satureoides* (100 mg/kg). (a) After 1 h, LPSs from *E. coli* 026:B6 (1 mg/2 mL) or PBS were injected directly into the air pouch, and 1 h later TLR-4 expression was measured in blood leukocytes by flow cytometry. (b) Circulating leukocytes were collected and *in vitro* stimulated by PMA. DCFH formation was measured by flow cytometry. Data are expressed as mean ± S.E.M. of 5-6 animals in each group. Statistical analysis was performed using ANOVA followed by Tukey's test. *P < 0.05 and ***P < 0.001 versus vehicle; ^#^ *P* < 0.05 and ^###^ *P* < 0.001 versus sham.

To elucidate the molecular mechanism of the anti-inflammatory effect shown by *A. satureoides *extract, membrane TLR-4 expression was evaluated by flow cytometry. The results showed that neutrophils obtained from animals orally treated with *A. satureoides *extract showed lower TLR-4 expression than animals treated with vehicle (Figure 6(a)). The reduction of TLR-4 expression might be totally or partially responsible for the anti-inflammatory activities observed in this study and might also suggest that this mechanism is a unique pathway of *A. satureoides *extract action. To investigate this hypothesis, neutrophils collected from treated rats were stimulated with PMA *in vitro*. PMA is lipophilic and directly stimulates the phosphorylation of PKC kinases, which are responsible for activation of the respiratory burst [18, 19]. Neutrophils from animals treated with *A. satureoides *extract and incubated with PMA for 30 min demonstrated a significant reduction in the production of PMA-stimulated reactive oxygen species (ROS), as measured by DCFH formation (Figure 6(b)). 

### 3.8. *A. satureoides* Hydroalcoholic Extract Treatment Does Not Cause Systemic Toxicity

**Figure 7:**
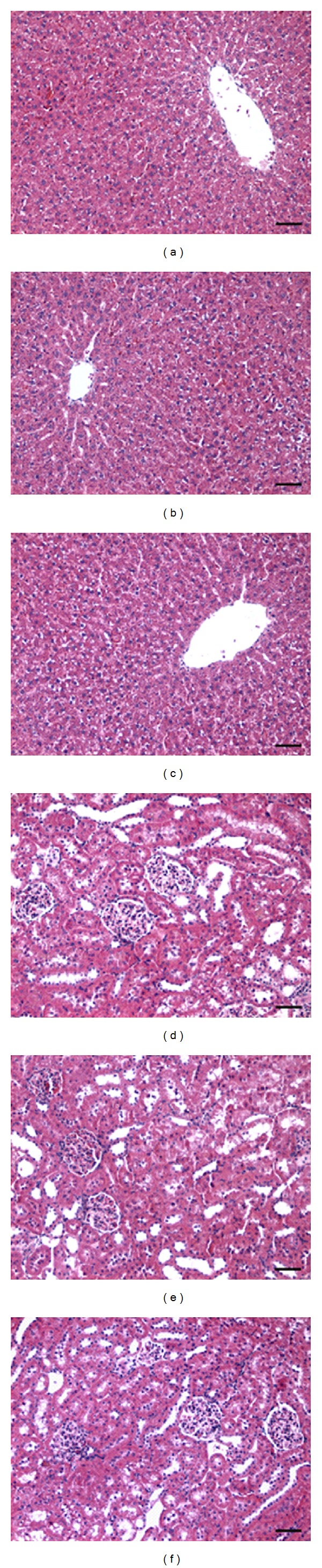
Effects of *A. satureoides* hydroalcoholic extract on liver and kidney histology in treated rats. Control (sham, air pouch induced without treatment), vehicle (PBS/ethanol 10%), and *A. satureoides* extract (100 mg/kg). There were no histological alterations on liver and kidney tissues. Hematoxylin-eosin. Scale bar = 20 *μ*m.

Liver and kidney biochemical and histological parameters were investigated in rats treated with vehicle or* A. satureoides*. Data showed that activities of the main hepatic enzymes, AST, ALT, and gamma-GT, as well as levels of creatinine and urea were equivalent in samples collected from rats treated with *A. satureoides *hydroalcoholic extract or vehicle (Table 2). Furthermore, no alteration in liver and kidney structures was observed in animals from both treatments (Figure 7).

## 4. Discussion

Neutrophils exert an important role in the induction of innate inflammatory reactions and in the transition between innate and immune responses. Therefore, in the case of exacerbated reactions, blockage of their functions represents a therapeutic strategy. Here, we show that *in vivo* treatment with *A. satureoides *inflorescence hydroalcoholic extract significantly impaired neutrophil migration into LPS-induced inflamed exudates, and effects on neutrophil migratory properties and secretion of chemotactic mediators might be involved in its anti-inflammatory action. Although expression of TLR-4, the main receptor for LPS binding, was reduced on the neutrophil membrane and might be responsible for the activity of *A. satureoides *extract in these cells, other forms of action might be involved, as a lower oxidative burst was observed following direct intracellular activation of PKC kinases in neutrophils. 

 Much evidence has shown that plant extracts containing flavonoids exert anti-inflammatory effects [20–24]. Previous studies have shown that *A. satureoides *extracts contain luteolin and quercetin [1, 2, 8, 9], and here, HPLC analysis corroborated that the two flavonoids are the main compounds within the inflorescence extract; however, GC-MS analysis showed that the *A. satureoides *extract also contains steroids and fatty acids. Based on the anti-inflammatory effects of flavonoids, it is expected that *A. satureoides *inflorescence extract can reduce neutrophil influx. Here, we show for the first time that *A. satureoides* extract reduces *in vivo* LPS induced neutrophil migration, which might relate to reduced TLR-4 membrane expression and therefore corroborate the action of quercetin and luteolin on TLR-4 expression [25–28]. LPS treatment induced TLR-4 expression, as visualized by enhanced membrane density in cells collected from vehicle-treated animals in comparison to those obtained from sham animals. TLR-4 expressions on cells collected from extract-treated rats were reduced. However, it is notable that reduced TLR-4 is not the only pathway of action of *A. satureoides *extract on neutrophils, as neutrophils collected from rats treated with *A. satureoides *extract showed a reduced oxidative burst elicited by *in vitro* PMA stimulation, which directly activates PKC phosphorylation and phagocyte NADPH oxidase, leading to the release of reactive metabolites [29].

 Data in our study do not show the direct effect of *A. satureoides *extract on neutrophil function, as *in vitro* neutrophil incubation with hydroalcoholic extract caused necrosis, even at low concentrations (data not shown). Cell death was not detected in vehicle-incubated cells, excluding the action of alcohol in the toxic effect (data not shown). It is relevant here that cytotoxicity caused by *in vitro* incubation with flavonoids is not cited in the literature. Many *in vitro *experimental studies have shown the direct action of extracts containing flavonoids on neutrophil function, but this has not been associated with cell death [30–33]. It is possible that the experimental conditions and technical approach to quantify cell death are responsible for divergent results. In the present study, flow cytometry quantification of Annexin-V- and PI-labeled neutrophils was used to detect cell membrane alterations, as modifications of the cell surface structure also alter the adhesion and locomotory response of neutrophils. Notably, *in vivo* extract treatment did not alter neutrophil viability, and subsequent assays were performed using this experimental strategy. Kinetic studies will be carried out to investigate the mechanisms in the absence of extract toxicity *in vivo*. In addition, the lack of functional or morphological alterations in kidney and liver following *A. satureoides *extract treatment strongly supports the absence of *in vivo* toxicity.

 As previously mentioned, neutrophil migration is dependent on initial contact of neutrophils with the endothelial cells from postcapillary venules [14], and intravital microscopy assays allow the visualization of leukocyte behavior in the microcirculatory network [34–36]. Data here clearly show that *in vivo* administration of the *A. satureoides *extract reduced the number of rolling and adhered leukocytes in LPS-stimulated mesentery. These data were further corroborated by the altered number of L-selectin- and *β*2-integrin-positive neutrophils, suggesting that *in vivo* extract treatment modifies the adhesive properties of neutrophils to the endothelium, which then impairs their migration into inflamed tissue. It has been shown that genetic deficiency of L-selectin or *β*2-integrin on leukocyte membranes reduces cell influx into inflamed areas, leading to accumulation of neutrophils in the blood [37–39].

Leukocyte adhesion to endogenous substrates, such as endothelium and extravascular matrix constituents, and direct migration to the damage site are dependent on the interaction between chemotactic mediators and specific receptors, mainly those expressed on cell membranes which activate pathways involved in adhesion and migration [40–43]. In this context, leukotriene B4 and CINC-1 are chemoattractants secreted by different cells in the inflammatory process, including migrated neutrophils, resident macrophages, mast cells, and fibroblasts [44–48]. Data in this study show that *in vivo* treatment with *A. satureoides *extract reduced the amount of both mediators in the inflammation exudates, showing the ability of the extract to inhibit the secretion of inflammatory cells. The cells that are responsible for this reduced secretion have been not established; nevertheless, *A. satureoides *treatment might inhibit the secretory activity of resident cells in the subcutaneous tissue, as the reduced levels of chemoattractants were similar in exudates collected 1 or 4 h after LPS injections, irrespective of the number of neutrophils in the pouches.

 Taken together, data presented here show the mechanisms of *A. satureoides *inflorescence extract on neutrophil influx using *in vivo* approaches, which might be responsible for the ethnopharmacological application of the extract. This might also be the mechanism involved in antiulcerogenic activity of the extract [2], as neutrophil influx into damaged stomachs is a hallmark of acute gastric disease. 

## 5. Conclusions

Taken together, data presented here from different *in vivo* studies show the mechanisms of the anti-inflammatory effect of *A. satureoides *hydroalcoholic extract. Based on these findings, we have highlighted the inhibitory actions of* A. satureoides *hydroalcoholic extract on adhesive and migration properties, TLR-4 expression, and oxidative metabolism of neutrophils, which might contribute to its anti-inflammatory effects and help to explain the use of *A. satureoides *extract as a therapeutic agent.

## Figures and Tables

**Table 1:** Effects of *A. satureoides* hydroalcoholic extract on *in vivo* cell viability.

Treatment	Dose	ALT	AST	Gama-GT	Urea	Creatinine
	(mg/kg)	(mg/dL)	(mg/dL)	(mg/dL)	(mg/dL)	(mg/dL)
Sham	—	37.65 ± 1.96	124.60 ± 9.46	9.04 ± 2.20	37.02 ± 2.83	0.53 ± 0.08
Vehicle	—	41.60 ± 2.57	113.60 ± 17.45	3.25 ± 2.34	33.59 ± 0.83	0.53 ± 0.41
*A. satureoides *	100	35.07 ± 1.92	106.60 ± 10.63	5.44 ± 2.18	37.20 ± 2.57	0.35 ± 0.06

Data are expressed as mean ± S.E.M. of 6 animals in each group. Sham (air pouch induced without treatment). Statistical analysis was performed using ANOVA followed by Tukey's test.

**Table 2:** Effects of *A. satureoides* hydroalcoholic extract on biochemical parameters.

Treatment	Dose	Apoptotic	Later apoptotic	Necrotic	Viable
	(mg/kg)	cells (%)	cells (%)	cells (%)	cells (%)
Sham	—	2.23 ± 0.39	6.90 ± 0.69	13.13 ± 1.79	70.12 ± 5.55
Vehicle	—	1.31 ± 0.14	3.77 ± 0.19	12.45 ± 1.15	82.71 ± 1.38
*A. satureoides *	100	0.82 ± 0.05	7.32 ± 0.56	19.13 ± 1.25	74.01 ± 2.38

Data are expressed as mean ± S.E.M. of 6 animals in each group. Sham (air pouch induced without treatment). Statistical analysis was performed using ANOVA followed by Tukey's test.
